# De Abortu

**Published:** 1837

**Authors:** 


### Art. VIII.—*De Abortu.*

Several abortions and still borns from one *matrix*, are now on our table, in a state of preservation; and give new proof, that one miscarriage is generally followed by more. The first of this progeny was christened, "The Western Medical Gazette; a semi-

MONTHLY JOURNAL, devoted to Medicine and the Collateral Sciences;" Cincinnati, Ohio. It was delivered by four *obstetrices*, on the first of January, 1833; and had enstamped on its forehead, an immense *nævus maternus*, resembling the "*Ohio Medical College.*" It manifested signs of vitality for about ten months.

The second appeared early in 1834, and received a similar name. A *smith* forged a pair of forceps to aid the *accoucheuses*. Being born without the *nævus*, it seemed to have a better chance of living than the last. It was soon found, however, to labor under *tabes mesenterica infantilis*, and was cast away among the *reeds*, where it was fished up and devoured by a *drake*.

In the succeeding year, 1835, *Alma Mater* was again *in stramento*. Her chief *obstetrix* was a *mason*, and the cognomen of her offspring, "THE OHIO MEDICAL REPOSITORY." It gasped but four times, and expired.

Rest for the *apparatus gestationis* now seemed advisable, and 1836, was a year of sterility and recuperation. To make amends for the loss of time, 1837 was signalized by a *fœtus*, which from its great size, *seemed* to have attained to maturity. The *abortio* continued from January to June, and all her professors assisted in the *accouchement*. As it is unlucky to name one child after another, which has died, this was christened, "THE WESTERN QUARTERLY JOURNAL OF PRACTICAL MEDICINE." High hopes now filled the hearts of all the gossips; but it was soon discovered, that the child was in fact asphyxiated, and still worse, that its mother had been seriously crippled by the severity of the labor; whereupon a majority of her attendants abandoned her to her fate, and put off for employment elsewhere.

During the four years, of which we have written her parturient biography, she had, also, one *extra uterine* conception, of which she was delivered by an unknown hand. It was little better than a mere *mola*, being deformed, squalid and rickety, but manifested all the antipathies and attachments, which characterized the family. It was dubbed the "OHIO MEDICAL REFORMER." The regular man-midwives, wished, for the honor of *Alma Mater*, that it should be regarded as an example of SPONTANEOUS GENERATION; but its temper betrayed its consanguinity with the legitimates, and it was, moreover, brought forth in the same nursery.

We owe an apology to all concerned, for not having registered these miscarriages, at an earlier day; but hope the faithful manner in which the work is now done, will atone for past negligence. The names and ages of the whole progeny, are here accurately set down, that generations to come, may sorrow over their "untimely fate." How much those generations have lost, it would be as difficult for us to make out, as it would, the loss which the world has suffered, by the death of Paracelsus, before the successful concoction of his *elixir vitæ.* But the question may, perhaps, stir up the genius of posterity, to whom, therefore, we transmit the *data*, here for the first time brought together; and shall conclude by inscribing on the cenotaph of these beautiful *vituli abortivi*—SIC INGENIUM MUNDI TRANSIT. D.

